# Bioinformatics Analysis of Peroxisomal Biogenesis Factor Proteins in Breast Malignancy for Introducing Potential Prognostic Biomarkers 

**DOI:** 10.30699/ijp.2025.2030953.3311

**Published:** 2025-03-10

**Authors:** Nima Mahdei Nasirmahalleh, Mina Hemmati, Negin Parsamanesh

**Affiliations:** 1 *Student Research Committee, Department of Medical Biochemistry, School of Medicine, Zanjan, Iran*; 2 *Department of Clinical Biochemistry, School of Medicine, Zanjan University of Medical Sciences, Zanjan, Iran*; 3 *Zanjan Metabolic Diseases Research Center, Zanjan University of Medical Sciences, Zanjan, Iran*; 4 *Department of Genetics and Molecular Medicine, School of Medicine, Zanjan University of Medical Sciences, Zanjan, Iran*

**Keywords:** Peroxine, Breast Tumor, Prognostic Marker

## Abstract

**Background & Objective:**

Breast cancer (BC) is the most common type of malignant neoplasm and is the primary cause of mortality among women aged 45 to 55 years. Studies indicate that cancer displays irregular metabolic patterns in contrast to normal tissue. Furthermore, there is compelling evidence supporting the significant role of peroxisomes in the intricate metabolic processes of cancer. Peroxisomal biogenesis factors (PEXs), which are peroxisomal proteins, control activities such as the degradation and biogenesis of peroxisomes. Hence, the correlation between peroxisomal biogenesis factor expression and BC was explored, to introduce key proteins and potential biomarkers by analyzing.

**Methods:**

This study utilized UALCAN, GenExMiner v4.8, Metascape, STRING, TIMER, the Kaplan-Meier plotter, The Human Protein Atlas, MirTarBase, and cBioportal.

**Results:**

The transcriptional levels of PEX6/7/10/11B/13/16 in BC tissues were significantly elevated, whereas the transcriptional levels of PEX2/3/5/11A/12/19 were significantly reduced. High expression levels of PEX 2/3/10/12/11G /26/13/16/14 were significantly related to shorter relapse-free survival, and higher mRNA expression of PEX 11B/11G/11A/12/19 was significantly associated with longer overall survival of BC patients. We identified has-miR-4318 and has-7106-3p as more correlated miRNAs with the PEX family.

**Conclusion:**

Our results may provide novel insights for the selection of therapeutic targets and prognostic biomarkers for BC.

## Introduction

Cancer is considered to be a prominent contributor to mortality and a significant impediment to the advancement of life expectancy across nations (1). Among the various types of cancer, breast cancer (BC) stands as the most prevalent form of malignant neoplasms (2). And in women between the ages of 45 and 55, it is considered the most important cause of death (3). Also, it is the second leading cause of death from cancer (4). Based on the evaluations presented by the Global Cancer Observatory in 2020, there was a notable emergence of 19.3 million new cases of cancer in the same year, alongside an estimated 10.0 million deaths linked to cancer (5). It has been projected that breast cancer will be the prevailing form of cancer in the year 2040, affecting approximately 364 000 individuals (4). Various risk factors, including sex, aging, estrogen levels, family history, genetic mutations, and unhealthy lifestyle choices, can significantly increase the probability of breast cancer advancement on a global scale (6, 7). The employment of the immunohistochemical method within the defined boundaries of the laboratory, verified by the highly regarded American Society of Clinical Oncology, has indisputably confirmed that the existence of human epidermal growth factor receptor 2 (HER2), estrogen (ER) and progesterone (PR) is universally perceived among individuals affected by the formidable and widespread forms of breast cancer that demonstrate both aggressive and metastatic attributes (8). 

The rise of high-throughput next-generation genetic sequencing and progressions in molecular methodologies have presented findings suggesting that cancer displays atypical metabolic behavior when contrasted with healthy tissue (9). Recently, an increasing body of evidence has emerged, pointing toward the presence of a growing reservoir of information that strongly implies the active involvement of peroxisomes in the intricate dynamics of cancer, a complex ailment characterized by a disruption in the normal metabolic processes. Enzymes involved in the processing of lipids within peroxisomes exhibit increased levels in various types of tumors, including breast cancer (9-11). Ischemia/reperfusion injury could result in systemic inflammatory reaction syndrome or numerous organ dysfunction syndrome; there is still a long way to progress therapeutic outcomes (12). 

The peroxisome serves as a metabolic platform for diverse cellular constituents, encompassing the production of bile acid and ether phospholipid, the breakdown of purine, and the oxidation of elongated fatty acid chains (13, 14). Peroxisomes exhibit intriguingly dual roles in both the production and removal of reactive oxygen species (14, 15). The subsequent occurrence of deregulation in the dynamics of peroxisomes has been postulated as a potential factor contributing to its role in promoting tumorigenesis (16, 17). It was noted and recorded through empirical observation that a direct and unmistakable correlation exists between the level of activity exhibited by specific enzymes located within the peroxisomal structures and the histopathological grade that the tumor in question has attained. This discovery strongly implies that peroxisomes can be considered a viable and effective means by which the process of grading tumors can be accomplished, thereby shedding light on an important aspect of tumor analysis and evaluation (18). 

Peroxins (PEXs), which are peroxisomal proteins, control activities such as the degradation and biogenesis of peroxisomes (19, 20). Fourteen distinct PEX genes, constituting a vast array of genetic information, are responsible for encoding peroxins. These peroxins, in turn, play a crucial role in facilitating the intricate process of peroxisome assembly. Among their manifold functions, these peroxins govern the importation of both peroxisomal matrix proteins and membrane proteins, effectively ensuring the seamless integration and organization of these vital components within the peroxisomal structure (21). 

Bioinformatics analysis has garnered increased interest among researchers as a potential avenue for discovering innovative biomarkers in various forms of human cancer (22, 23). Therefore, the limited information regarding the association between PEX family genes and BC necessitates our undertaking an investigation into the various roles played by PEX family genes to identify potential prognostic markers. This investigation will involve an in-depth analysis of PEX family expression in BC, which will be accomplished by leveraging the powerful data mining capabilities of UALCAN, TIMER, and Kaplan-Meier plotter databases. As a subsequent step, we will delve into the intricate relationship between PEX expression and the levels of immune infiltrating abundance using the comprehensive resources offered by the TIMER database. This will enable us to identify and introduce a range of molecular biomarkers and key proteins that are closely linked to BC.

## Materials and Methods

### Evaluation of Prognostic Values of PEX Family

In order to assess the predictive feature of PEX family member expression, the Kaplan-Meier plotter was utilized (http://kmplot.com/analysis).(24.(24)) within the entirety of individuals diagnosed with breast cancer. Previously, investigators presented investigations on the survival rates, encompassing overall survival (OS) and relapse-free survival (RFS). Typically, the log-rank *P* value with a threshold of less than 0.05 was customary to determine significance. 

### Relationship of PEX Expression With Clinicopathological Characteristics of BC

In order to determine the relationship between PEX expression and the clinicopathological features of breast cancer, we utilized the bc-GenExMiner v4.8 software tool (http://bcgenex.ico.unicancer.fr/BC-GEM), which includes variables like human epidermal growth factor 2 (HER2), progesterone receptor (PR), tissue age nature, nodal status, and estrogen receptor (ER). The disparity in PEX mRNA expression among individuals diagnosed with breast cancer, in conjunction with various molecular and clinical factors, was evaluated utilizing the Dunnett-Tukey-Kramer's and Welch's statistical tests. A *P* value of less than 0.05 was regarded as highly significant. 

### mRNA Expression Analysis of PEX Family

GEPIA (http://gepia.cancer-pku.cn/) is a tool that provides statistics about RNA sequence expression in normal and tumor samples. From this database, which was developed in Peking University (25), the differential mRNA expression of genes PEX was analyzed. The *P* value cutoff considers significantly less than 0.05. 

### Transcriptional Expression of the PEX Family in BC

We used UALCAN (http://ualcan.path.uab.edu/analysis.html) to investigate the relative transcript expression of the PEX family in different stages of BC (26). This database contains studies according to the Cancer Genome Atlas (TCGA). P < 0.05 was significant. 

### PEX Family and Their Ligands Interaction

The system evaluates, consolidates, and incorporates publicly accessible protein-protein interaction (PPI) repositories and enhances them with statistical forecasts of possible functionalities within the STRING database (https://string-db.org/).(27.(27)). We used this database to complete a PEX network study of PEX family members and their relationships. 

### Relationship of PEX Expression With Immune Infiltrating Cells

We utilized the TIMER database (https://cistrome.shinyapps.io/timer/) (28) to determine the relationship between the expression of PEX and immune infiltrating cells (dendritic cell, CD4+ T cells, CD8+ T cells, macrophages B cells, and neutrophils). 

### Protein-Protein Interaction Network

GeneMANIA database (http://www.genemania.org) (Dorner et al., 2011) was used to find data on protein-gene interaction, coexpression, pathway, coexistence, and similarity of protein regions of transferred genes. We used this database for the protein-protein interaction relationships of PEX family members and their relationships. 

### Gene Enrichment Analysis

Enrich R (https://maayanlab.cloud/Enrichr/) is a common tool for gene annotation and gene sequence enrichment studies (29). According to the gene list working paper, the "Express Analysis" function was used to determine the enrichment of the PEX family and neighboring genes. Similarly, “functional enrichment” including KEGG and GO pathways (eg, CC: cellular component, MF: molecular function and BP: biological process) was performed on PEX members and P value < 0.05 was significant. 

### Protein Expression Patterns of PEXs in BC

The Human Protein Atlas (https://www. proteinatlas.org/about/licence) is a website that integrates many omics technologies, including genome-wide proteomics, mass spectrometry, systems biology, and immunohistochemistry to provide information on immunohistochemistry. (Asplund et al., 2012). Our study used a direct difference in protein expression of PEX family members between tumor and normal BC tissue using immunohistochemical imaging. 

### Exploration of Multidimensional BC Genomics Data Sets

We use publicly available data generated from the cBioportal web platform (https://www.cbioportal.org/). We selected invasive breast cancer (TCGA, Firehose Legacy, n = 960 patients) to study. Correlation plots showing the relationship between mRNA expression levels in the samples and gene copy numbers available through the cBioportal were analyzed. 

## Results

### Nine PEX Family Members' Expression Patterns Were Analyzed in Individuals Diagnosed With BC.

We used the UALCAN resource to investigate expression differences of 16 PEX genes between normal and tumor tissues of BC patients at a transcriptional level. Our results showed that the mRNA expression of PEX 6/7/10/11B/13/16 is increased in tumor tissue compared with normal tissue (PEX6, *p* =<0.0001; PEX7, *p* =<0.0001; PEX10, *p*=<0.0001; PEX11B, p=<0.0001; PEX13, p=<0.0001; PEX16, p=<0.0001).(Fig1A) Whereas PEX2/3/5/11A/12/19 mRNA levels were lower (PEX2, p=<0.0001; PEX3, p=<0.0001; PEX5, p=<0.0001; PEX11A, p=<0.0001; PEX12, p=<0.0001 and PEX19, p=<0.0001). (Fig1B) The transcriptional level of PEX1/14/26 was not significantly different between BC and normal tissues.

**Fig.1A:**
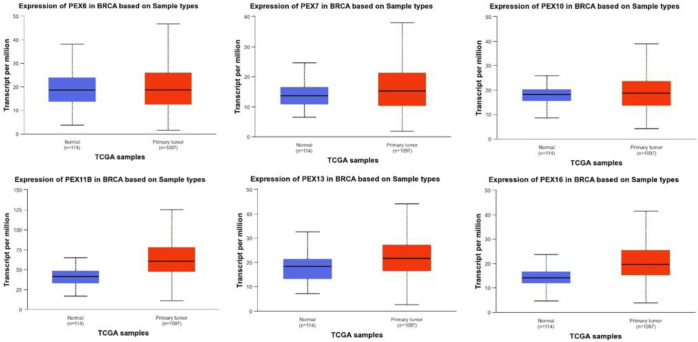
The transcription of PEX genes in BC (UALCAN). The transcriptional levels of PEX 6/7/10/11B/13/16 in BC tissues were elevated.The p-value was <0.05

**Fig.1B:**
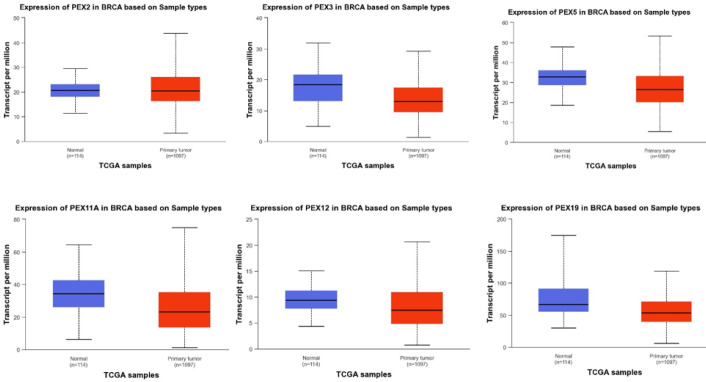
The transcription of PEX genes in BC (UALCAN). the transcriptional levels of PEX2/3/5/11A/12/19 were reduced. The p-value was <0.05

**Fig. 2:**
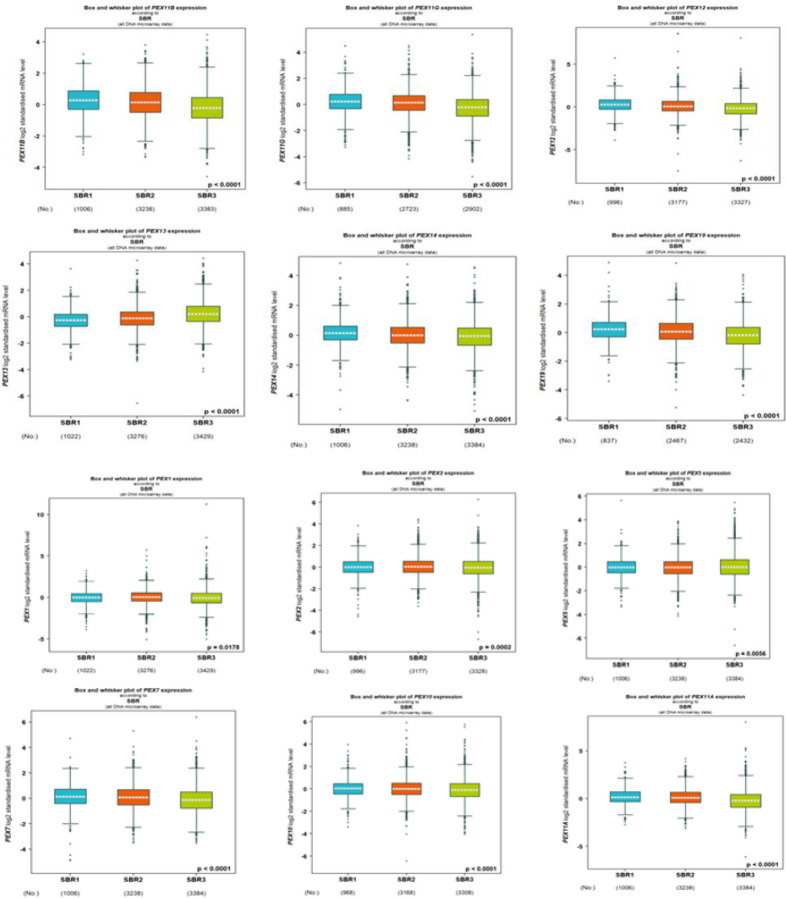
The relationship between PEXs expression and SBR grade of BC patients (bc-GenExMiner).

**Fig. 3:**
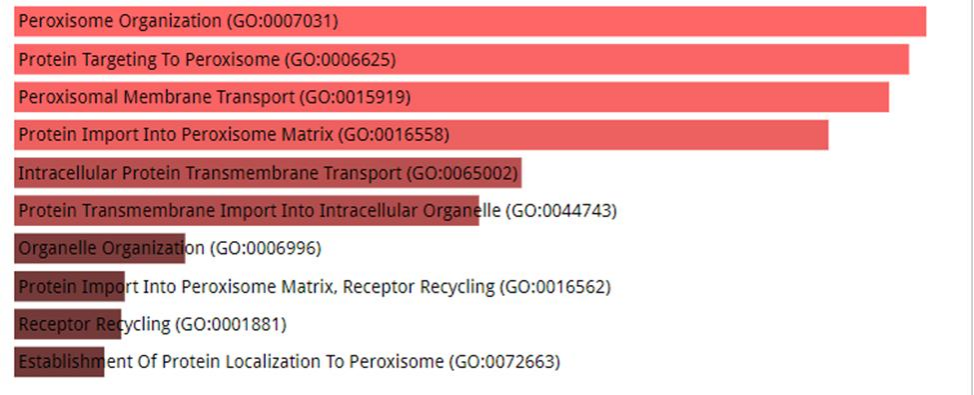
The examination of the PEXs family in breast cancer through enrichment analysis (Enrichr) is conductedThe GO enrichment in biological process.

### Association Between the mRNA Expression Levels of PEXs and the Clinicopathological Features of BC Patients.

To assess the correlation between PEX mRNA expression and clinicopathological characteristics of breast cancer, bc-GenExMiner was utilized for the analysis of the association between PEX expression and SBR score in breast cancer patients. The data presented in Figure 2 illustrates that a higher SBR grade was linked to decreased mRNA levels of PEX1/2/5/7/10/11A/11B/11G/12/13/14/19/26 (p< 0.05). As indicated in Table 1, we identified a downregulation of PEX1 (P=0.0153), PEX2 (P<0.0001), PEX3 (P<0.0001), PEX7 (P<0.0001), PEX11A (P=0072), PEX11G (P=0.0079), PEX12 (P<0.0001) and PEX19 (P<0.0001) expression in the <51 years old group compared to that in the >51. 

According to the results in Table 1, we identified a downregulation of PEX1 (p= 0.0153), PEX3 (p<0.0001), and PEX7 (p<0.0001), PEX11A (p= 0.0072), PEX11G (p= 0.0079), PEX12 (p= p<0.0001), PEX19 (p<0.0001) expression in the <51 years old group compared to that in the >51. 

We noted with interest that the mRNA expression level of PEX2 was found to be significantly reduced in the group aged over 51 years compared to those under 51 years (p<0.0001). 

As indicated in Table 1, the mRNA level of PEX3 (p=0.0106 & p=0.0497), PEX12 (p< 0.0001) and PEX26 (p< 0.0001 & p=0.0005) was lower in ER & PR-positive BC compared to ER & PR-negative BC; contrariwise, the mRNA levels of PEX1/7/11A/11B/11G/12/19 (p< 0.0001), PEX2 (P=0.0106 & P=0.0212), PEX10 (P<0.0001 & P=0.0026) was lower in ER & PR-negative group compared to ER & PR-positive. 

The mRNA level of PEX16 (p=0.0434) was lower just in the ER-negative group compared to the ER-positive. 

We observed that PEX11A (P=0.0002), PEX11G (p=0.0148), and PEX12 (p=0.0270) in the negative nodal status group were downregulated compared to the positive nodal status and PEX5 (p=0.0077) in the positive nodal status group were downregulated compared to the negative nodal status. 

### GO Term and KEGG Pathway Enrichment Analysis

Enrichment analysis of Gene Ontology (GO) terms and top 100 associated genes from the Kyoto Encyclopedia of Genes and Genomes (KEGG), PEX was obtained using “Enrichrand”. 

Package First analyzed the 100 correlated genes of PEXs for molecular function and biological process enrichment. 

These genes were found to have Peroxisome Organization (GO:0007031; P= 1.061e-37), Protein Targeting To Peroxisome (GO:0006625; P= 3.871e-37), Peroxisomal Membrane Transport (GO:0015919; P= 1.316e-36), Protein Import Into Peroxisome Matrix (GO:0016558; P= 8.109E-e31), Intracellular Protein Transmembrane Transport (GO:0065002; p= 1.128e-26), Protein Transmembrane Import Into Intracellular Organelle (GO:0044743, p= 1.821e-25), Organelle Organization (GO:0006996; p= 1.002e-17), Protein Import Into Peroxisome Matrix, Receptor Recycling (GO:0016562; p= 9.823e-16), Receptor Recycling (GO:0001881; p= 1.110e-15), Establishment Of Protein Localization To Peroxisome (GO:0072663; p= 3.437e-15). (Fig3.) Moreover, we constructed a protein-protein interaction (PPI) network for 16 PEX family members with their ligands using STRING databases to explore their potential interactions. 

As shown in Table 2, the PEX family network comprises protein-protein interaction and related genes. 

### Correlation of PEX Expression in BC With Infiltration of Immune Cells

**Fig.4:**
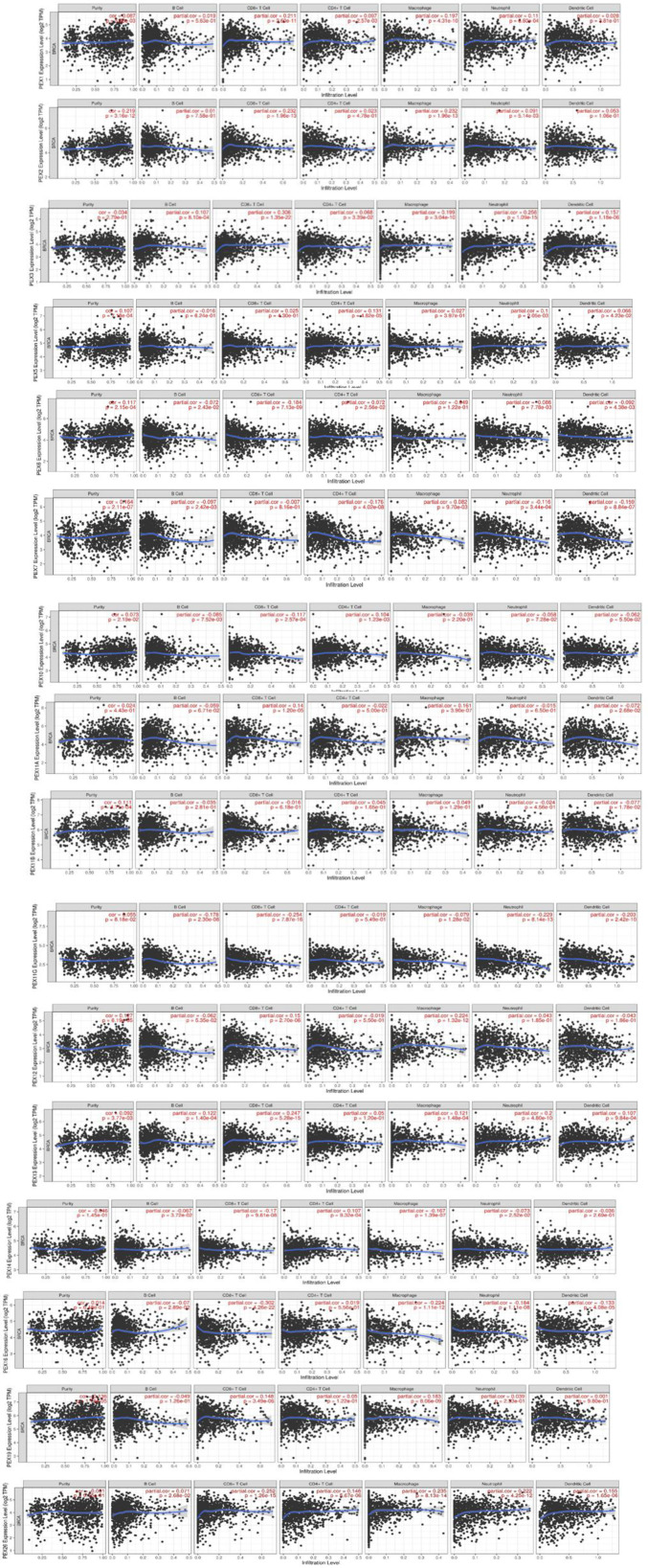
The association of PEX family with immune cell infiltration in the tumor immune microenvironment (TIME)

The present investigation utilizes the "Survival module" to establish the relationship between immune infiltration and the expression of PEX genes by employing the TIMER database (Figure 4). 

There was a positive and negative correlation between the expression of PEX genes and the infiltration of macrophages, neutrophils, dendritic cells, CD4+ T cells, B cells and CD8+ T cells in BC. 

The types of these correlations and their P values ​​are described in Table 3. 

5 Prognostic Analysis of PEXs in Patients With BC

**Fig 5:**
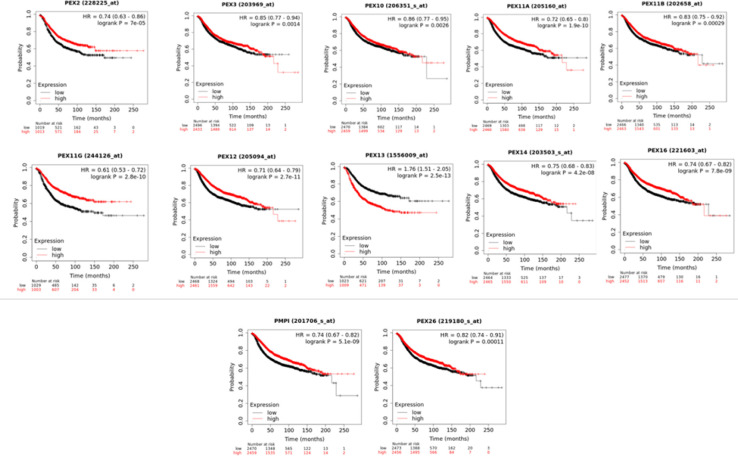
The association of mRNA expression of PEX family with relapse-free survival (RFS) of BC patients (Kaplan-Meier Plotter).

To assess the prognostic significance of PEX constituents in breast cancer, an examination was conducted to analyze the relationship between the expression of these genes and the clinical outcomes by utilizing the Kaplan-Meier (KM) database***.*** As relapse-free survival curves and log-rank test analyses are presented in Fig.5, high expression level of *PEX2 (*HR = 0.74, 95% CI: 0.63-0.86, *P* = 7e-05), *PEX3(*HR = 0.85, 95% CI:0.77-0.94, *p* = 0.0014), *PEX11A (*HR = 0.72, 95% CI: 0.65–0.8, *P *= 1.9e-10), *PEX10 (*HR = 0.86, 95% CI:0.77-0.95, *p* = 0.0026), *PEX11B* (HR = 0.83, 95% CI:0.75-0.92, *p* = 0.0029), *PEX12 (*HR = 0.71, 95% CI:0.64-0.79, *p* = 2.7e-11), *PEX11G (*HR = 0.61, 95% CI: 0.53–0.72, *P* =2.8e-10) *PEX26 ( *HR = 0.82, 95% CI: 0.74–0.91, *P* = 0.00011), *PEX13 *(HR = 1.76, 95% CI: 1.51-2.05, *P* =2.5e-13) *PEX16 (*HR = 0.74, 95% CI: 0.67–0.82, *P* =7.8e-09) *PEX14 ( *HR = 0.75, 95% CI: 0.68–0.83, *P* =4.2e-08) and *PEX19(PMPI) ( *HR = 0.74, 95% CI: 0.67–0.82, *P* =5.1e-09) ) were significantly related to shorter relapse-free survival (RFS) of BC patients. 

**Fig 6:**
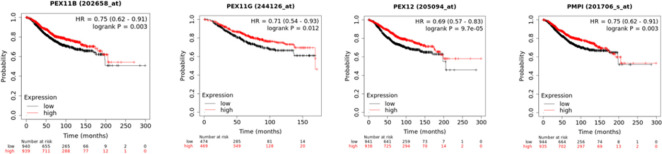
The association of mRNA expression of PEX family with overall survival (OS) of BC patients (Kaplan-Meier Plotter).

Interestingly, higher mRNA expression of PEX 11B/11G/11A/12/19 (PEX11B: HR = 0.75, 95% CI: 0.62-0.91, P = 0.03; PEX11G: HR = 0.71, 95% CI:0.54-0.93, p = 0.012; PEX11A: HR = 0.81, 95% CI: 0.67–0.98, P = 0.029; PEX12: HR = 0.69, 95% CI:0.57-0.83, p = 9.7e-05; PEX19: HR = 0.75, 95% CI:0.62-0.91, p = 0.003;) was significantly associated with longer overall survival (OS) of BC patients (Fig6.). 

These results suggested that mRNA expression level of *PEX11A/11B/11G/12/19 *plays a major role in breast cancer patients' prognosis and also, they may be used as novel and potential prognostic biomarkers for BC patients' survival. 

### Protein Expression Patterns of PEXs in BC

We analyzed the protein expression patterns of PEXs in BC patients using the Human Protein Atlas (HPA) database. 

### Identification of Key miRNAs Related to PEX Member's Family

This study identified microRNAs associated with the PEX family using the MirTarBase online database. 

(Table 4) After a database search, ten miRNAs were identified as the main miRNAs of the PEX family. 

As shown in Table 4, hsa-miR-4318 and hsa-miR-4680-3p were found to be miRNAs with a higher correlation with the PEX family. 

### Data Analysis from Patients with Breast Cancer from cBioPortal

We queried Breast Invasive Carcinoma (TCGA, Firehose Legacy, n = 960 patients) found evidence of PEX1, PEX2, PEX3, PEX5, PEX6, PEX10, PEX7, PEX11A, PEX11G, PEX11B, PEX13, PEX12, PEX16, PEX14, PEX26, PEX19 (Figure8) 7%, 18%, 5%, 7%, 5%, 5%, 5%, 6%, 21%, 6%, 8%, 6%, 7%, 5%, 17% and 6% respectively among the patients of BC. 

**Fig. 7:**
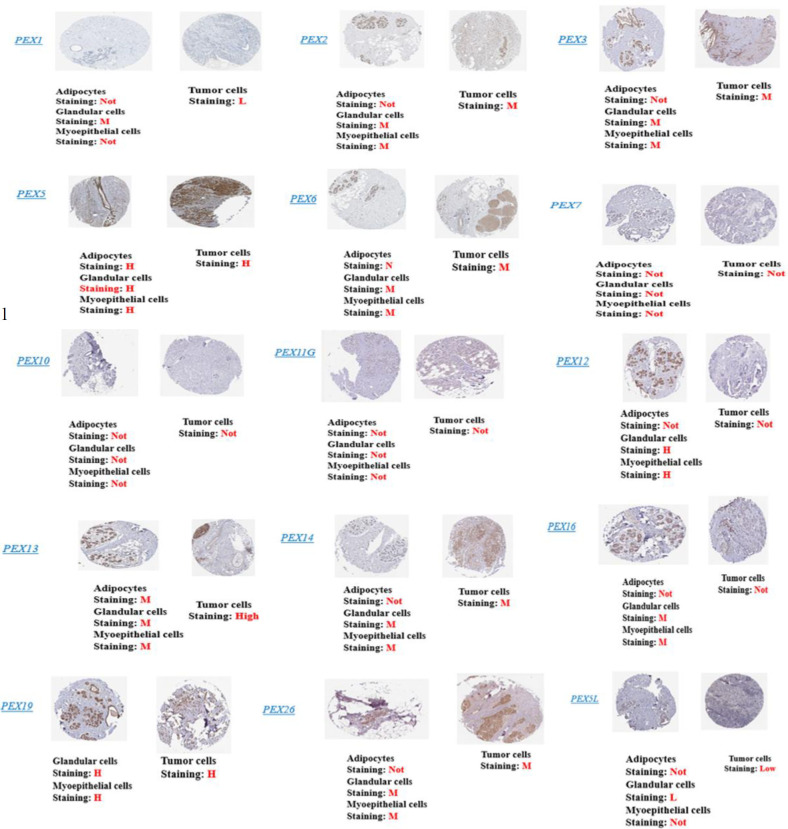
Representative Immunohistochemistry images of PEX family members in BC tissues and normal tissues (Human Protein Atlas Database). Left picture: normal, right picture: tumor. L: Low; M: Medium; None: Not detected, H: Hjgh.

**Table 1:** The relationship between mRNA level PEXs and clinicopathological features of BC patients (bc-GenExMiner v4.2).

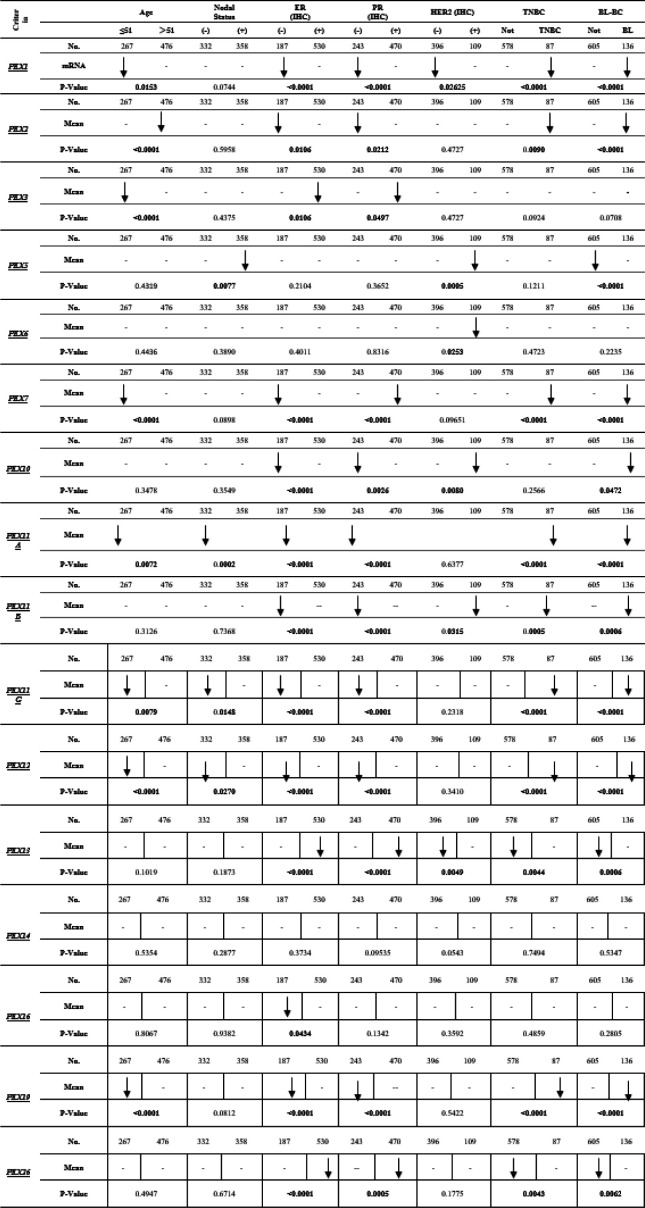

**Table 2:** Protein-protein interaction network of PEX family members by string databases

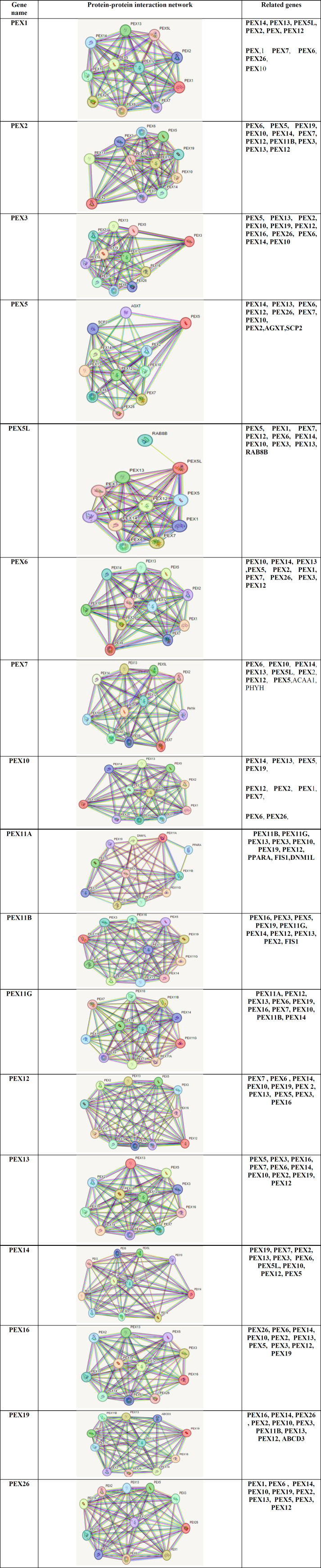

**Table 3:** positive and negative correlation between PEX genes expression and macrophage, neutrophil, dendritic cell, CD4+ T, B cells and CD8+ T cells infiltration in BC.

**macrophage**	dendritic cell	neutrophil	CD4+ T	B cells	CD8+ T cells	**macrophage**	dendritic cell	neutrophil	CD4+ T	B cells	CD8+ T cells	
**P-value**	correlation	P-value	correlation	P-value	correlation	P-value	correlation	P-value	correlation	P-value	correlation	
**4.31e-10**	0.197	2.81e-01	0.028	6.80e-04	0.11	2.57e-03	0.097	5.63e01	0.019	2.60e-11	0.211	PEX1
**1.96e-13**	0.232	1.06e-01	0.053	5.14e-03	0.091	4.78e-01	0.023	7.58e01	0.01	1.96e-13	0.239	PEX2
**3.04e-10**	0.199	1.18e-06	0.157	1.09e-15	0.256	3.39e-02	0.068	8.10e04	0.107	1.35e-22	0.306	PEX3
**3.97e-01**	0.027	4.23e-02	0.066	2.05e-03	0.1	4.82e-05	0.131	6.24e01	-0.016	4.30e-01	0.025	PEX5
**1.22e-01**	-0.049	4.38e-03	-0.092	7.78e-03	0.086	2.56e-02	0.072	2.43e-02	-0.072	7.13e-09	-0.184	PEX6
**9.70e-03**	0.082	8.84e-07	-0.15	3.44e-04	-0.116	4.02e-08	-0.176	2.42e-03	-0.097	8.16e-01	-0.007	PEX7
**2.20e-01**	-0.039	5.5e-02	-0.062	7.28e-02	-0.058	1.23e-03	0.104	7.52e-03	-0.085	2.912.57e-04	-0.117	PEX10
**3.90e-07**	0.161	2.68e-02	-0.072	6.50e-01	-0.015	5.00e-01	-0.022	6.71e-02	-0.059	1.2e-05	0.14	PEX11A
**1.29e-01**	0.049	1.78e-02	-0.077	4.56e-01	-0.024	1.65e-01	0.045	2.81e-01	-0.035	6.18e-01	-0.016	PEX11B
**1.28e-02**	-0.079	2.42e-10	-0.203	8.14e-13	-0.229	5.49e-01	-0.019	2.30e-08	-0.178	7.87e-16	-0.254	PEX11G
**1.32e-12**	0.224	1.86e-01	-0.043	1.85e-01	0.043	5.50e-01	-0.019	5.35e-02	0.062	2.70e-06	0.15	PEX12
**1.48e-04**	0.121	9.84e-04	0.107	4.80e-10	0.2	1.20e-01	0.05	1.40e-04	0.122	5.28e-15	0.247	PEX13
**1.39e-07**	-0.167	2.69e-01	-0.036	2..52e-02	-0.073	9.32e-04	0.107	3.72e-02	-0.067	9.61e-08	-0.17	PEX14
**1.11e-12**	-0.224	4.08e-05	-0.133	1.11e-08	-0.184	5.56e-01	0.019	2.89e-02	-0.07	4.26e-22	-0.302	PEX16
**8.06e-09**	0.183	9.80e-01	0.001	2.33e-01	0.039	1.22e-01	0.05	1.26e-01	-0.049	3.49e-06	0.148	PEX19
**8.13e-14**	0.235	1.65e-06	0.155	4.25e-12	0.222	5.67e-06	0.146	2.68e-02	0.071	1.26e-15	0.252	PEX26

**Table 4:** The enrichment analysis of the CHDs family in BrCa (Enrichr).Key miRNA of PEX Family from miRTar base.

Index	name	p-value	Adjusted P-value	Odds ratio	Combined score	Genes
1	hsa-miR-937-3p	0.009313	0.2415	124.83	583.76	PEX6
2	hsa-miR-5708	0.01100	0.2415	104.02	469.12	PEX18
3	hsa-miR-8074	0.01603	0.2415	69.32	286.51	PEX2
4	hsa-miR-4318	0.001371	0.1742	42.16	277.93	PEX2, PEX10
5	hsa-miR-7106-3p	0.001543	0.1742	39.63	256.60	PEX16, PEX5L
6	hsa-miR-4680-3p	0.001587	0.1742	39.05	251.70	PEX2, PEX13
7	hsa-miR-554	0.01854	0.2415	59.41	236.91	PEX13
8	hsa-miR-6075	0.01938	0.2415	56.71	223.63	PEX26
9	hsa-miR-381-5p	0.02021	0.2415	54.24	211.61	PEX18
10	hsa-miR-4540	0.02438	0.2415	44.54	165.44	PEX13

## Discussion

 The manifestation of congenital anomalies that cause severe illnesses and often result in fatality highlights the significant contribution of peroxisomes to human well-being. Nevertheless, the involvement of peroxisomes in human health extends well beyond the comparatively uncommon hereditary peroxisomal maladies. Participation in nonmetabolic mechanisms like senescence, antiviral immunity, and carcinogenesis underscores the extensive impact of peroxisomes on human health (14).

There are 14 human PEX genes that encode peroxisome proteins involved in multiple phases of peroxisome biogenesis, encompassing peroxisome matrix protein input, membrane formation, and peroxisome proliferation (30, 31). 

Various forms of neoplasms manifest changes in the abundance and functioning of peroxisomes. The levels of peroxisomal proteins or the enzymatic activities involved in peroxisomal metabolism were significantly diminished in colon (32), breast (33), and hepatocellular carcinoma (HCC) (34). Disruption of peroxisome biogenesis may lead to the inhibition of peroxisomal metabolism. The onset of new peroxisome formation includes the interaction of PEX3 with either PEX16 or PEX14 on the surfaces of the endoplasmic reticulum or mitochondria (35). Subsequent to this, the membranes containing PEX3/PE16 or PEX3/PEX14 are released from the organelles in the form of budding vesicles (36).

A recent investigation reveals that the inhibition of PEX2, a peroxin responsible for the degradation of peroxisomes through autophagy (known as pexophagy), decreased the rate of liver cancer tumor expansion (9). PEX2 and other peroxins—namely, PEX5, PEX10, and PEX12—play a pivotal role in ensuring the survival of malignant cells and, consequently, emerge as prospective therapeutic targets for combating neoplastic disorders (14). Another investigation uncovered that the proteins PEX3, PEX16, and PEX19 safeguard lymphoma cells by counteracting cell death prompted by the histone deacetylase inhibitor. Consequently, these proteins facilitate the development of tumors (36).

## Conclusion

Our current study indicated that the changed expression of some *PEX* members was significantly associated with clinical cancer outcomes in breast cancer patients. However, further investigations are needed to evaluate the *PEX* members studied in detail.

## Data Availability

Data are available upon reasonable request from the corresponding author.
